# 17% Non‐Fullerene Organic Solar Cells with Annealing‐Free Aqueous MoO*_x_*

**DOI:** 10.1002/advs.202002395

**Published:** 2020-09-21

**Authors:** Hong Nhan Tran, Sujung Park, Febrian Tri Adhi Wibowo, Narra Vamsi Krishna, Ju Hwan Kang, Jung Hwa Seo, Huy Nguyen‐Phu, Sung‐Yeon Jang, Shinuk Cho

**Affiliations:** ^1^ Department of Physics and Energy Harvest Storage Research Center (EHSRC) University of Ulsan Ulsan 44610 Republic of Korea; ^2^ School of Energy and Chemical Engineering Ulsan National Institute of Science and Technology Ulsan 44919 Republic of Korea; ^3^ Department of Materials Physics Dong‐A University Busan 49315 Republic of Korea; ^4^ School of Chemical Engineering University of Ulsan Ulsan 44610 Republic of Korea

**Keywords:** annealing‐freemetal oxides, charge transport layers, curing‐freemetal oxides, metal oxides, polymer solar cells

## Abstract

A charge transport layer based on transition metal‐oxides prepared by an anhydrous sol–gel method normally requires high‐temperature annealing to achieve the desired quality. Although annealing is not a difficult process in the laboratory, it is definitely not a simple process in mass production, such as roll‐to‐roll, because of the inevitable long cooling step that follows. Therefore, the development of an annealing‐free solution‐processable metal‐oxide is essential for the large‐scale commercialization. In this work, a room‐temperature processable annealing‐free “aqueous” MoO*_x_* solution is developed and applied in non‐fullerene PBDB‐T‐2F:Y6 solar cells. By adjusting the concentration of water in the sol–gel route, an annealing‐free MoO*_x_* with excellent electrical properties is successfully developed. The PBDB‐T‐2F:Y6 solar cell with the general MoO*_x_* prepared by the anhydrous sol–gel method shows a low efficiency of 7.7% without annealing. If this anhydrous MoO*_x_* is annealed at 200 °C, the efficiency is recovered to 17.1%, which is a normal value typically observed in conventional structure PBDB‐T‐2F:Y6 solar cells. However, without any annealing process, the solar cell with aqueous MoO*_x_* exhibits comparable performance of 17.0%. In addition, the solar cell with annealing‐free aqueous MoO*_x_* exhibits better performance and stability without high‐temperature annealing compared to the solar cells with PEDOT:PSS.

## Graphical abstract

Annealing‐free solution‐processable aqueous MoO*_x_* are developed and applied in bulk‐heterojunction polymer solar cells based on non‐fullerene system PBDB‐T‐2F:Y6. The solar cells with aqueous MoO*_x_* exhibit higher efficiencies and better stabilities without high‐temperature annealing compared to the solar cells with PEDOT:PSS.

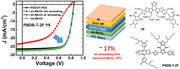

## 1 Introduction

Polymer solar cells (PSCs) based on organic semiconductors are considered the most promising candidate as a future mobile energy source due to their advantages, including light weight, low cost, flexibility, and simple fabrication process with the possibility of printable or roll‐to‐roll mass production.^[^ ^1^, ^2^, ^3^, ^4^, ^5^, ^6^ ^]^ The power conversion efficiency (PCE) of single junction polymer solar cells has steadily increased and recently reached over 16% due to the development of novel high quality donor and acceptor materials.^[^ ^7^, ^8^, ^9^, ^10^ ^]^ The increment of the efficiency of polymer solar cells was achieved by not only the development of higher performing new donor and acceptor materials, but also by improving the quality of charge transport layers (or called electrode buffer layers) such as the hole transport layer (HTL) and electron transport layer (ETL) inserted between the active layer and electrodes.^[^ ^11^ ^]^ It is clearly known that the inserted charge transport layers induce significantly improved efficiency by enhancing the charge extraction property and reducing leak currents which occur due to undesirable opposite charge flow. In addition, charge transport layers also have a significant influence on the stability of PSCs.

In earlier studies on polymer solar cells, poly(3,4‐ethylenedioxythiophene):poly(styrenesulfonate) (PEDOT:PSS) was the most popular hole transporting material because of its high conductivity and well‐matched work‐function (≈5.1 eV) for the highest occupied molecular orbital level of general donor polymers.^[^ ^12^, ^13^, ^14^, ^15^ ^]^ However, since it is known that the acidity of PEDOT:PSS may cause degradation of both the indium tin oxide (ITO) electrode and active layer,^[^ ^16^, ^17^, ^18^ ^]^ there has been extensive research on HTL materials to replace PEDOT:PSS. Among the several materials that can be utilized as an alternative to PEDOT:PSS, the most prominent candidate materials are transition metal oxides such as MoO_3_, V_2_O_5_, NiO, and WO_3_ due to their good charge transport properties and high stability.^[^ ^19^, ^20^, ^21^, ^22^ ^]^ In particular, MoO_3_ is the most widely utilized as an HTL in PSCs.

There are many ways to prepare the molybdenum oxide buffer layer, including a wet chemical sol–gel method, thermal evaporation, chemical vapor deposition, and pulsed laser deposition.^[^ ^23^, ^24^, ^25^, ^26^ ^]^ Normally, solution‐processable MoO*_x_* prepared by the sol–gel method has been mainly utilized for PSCs with a conventional structure. In the case of inverted PSCs, the thermal evaporation method is preferred for the deposition of the MoO*_x_* HTL. This is because of the necessity of a high temperature annealing step to achieve the desired hole transporting property in the case of solution‐processable MoO*_x_*. Unfortunately, normal organic‐based photovoltaic materials are not able to withstand high temperature annealing well. Moreover, the high‐temperature annealing process also acts as a disadvantage in the fabrication of flexible devices because general flexible substrates are not able to withstand high‐temperature annealing above 150 °C. Therefore, the development of annealing‐free solution‐processable metal oxides including MoO*_x_* is essential for the large‐scale commercialization of flexible PSCs.

Depending on the nature of the base solvent, the sol–gel method can be classified into two routes: an aqueous sol–gel method and anhydrous sol–gel method. The difference between these two routes is whether water is used as the base solvent or not. In the aqueous sol–gel route, water and alcoholic solvents are used as the reaction medium, while in the anhydrous sol–gel route, only organic solvents are used as the reaction medium.^[^ ^27^ ^]^ Typically, MoO*_x_* prepared by the anhydrous sol–gel method using MoO_2_(acac)_2_ is needed for high‐temperature curing to result in high quality. For example, Li and coworkers reported that MoO*_x_* HTL deposited by spin coating of a precursor solution prepared through the dilution of MoO_2_(acac)_2_ in anhydrous isopropanol is needed for high temperature annealing over 150 °C in air.^[^ ^28^ ^]^ Riedl et al. also reported that the MoO*_x_* film prepared by solution processing required high temperature annealing over 150 °C in an N_2_ atmosphere to obtain a high work function of 5.3 eV.^[^ ^29^ ^]^

In our present work, we developed annealing‐free solution‐processable “aqueous” MoO*_x_* (aq‐MoO*_x_*), and applied it in a bulk‐heterojunction (BHJ) PSC. An important reason for the high‐temperature annealing step after MoO*_x_* film deposition is to remove the ligands. We found that the acetylacetonate ligand of MoO_2_(acac)_2_ can be easily removed through the simple hydrolysis caused by a small amount of water. By adjusting the concentration of water in the sol–gel route for the MoO*_x_* precursor, we successfully developed an annealing‐free MoO*_x_* HTL with excellent electrical properties. The non‐fullerene solar cell based on a blend of poly[(2,6‐(4,8‐bis(5‐(2‐ethylhexyl‐3‐fluoro)thiophen‐2‐yl)‐benzo][1,2‐b:4,5‐b′]dithiophene))‐alt‐(5,5‐(1′,3′‐di‐2‐thienyl‐5′,7′‐bis(2‐ethylhexyl)benzo[1′,2′‐c:4′,5′‐c′]dithiophene‐4,8‐dione) (PBDB‐T‐2F, also called PM6) and non‐fullerene acceptor (2,2′‐((2Z,2′Z)‐((12,13‐bis(2‐ethylhexyl)‐3,9‐diundecyl‐12,13‐dihydro‐[1,2,5]thiadiazolo[3,4‐e]thieno[2,“3”:4′,5′]thieno[2′,3′:4,5]pyrrolo[3,2‐g]thieno[2′,3′:4,5]thieno[3,2‐b]indole‐2,10‐diyl)bis(methanylylidene))bis(5,6‐difluoro‐3‐oxo‐2,3‐dihydro‐1H‐indene‐2,1‐diylidene))dimalononitrile) (Y6) with the general MoO*_x_* layer prepared by the anhydrous sol–gel method showed a poor efficiency of 7.7% without annealing. If this anhydrous MoO*_x_* (an‐MoO*_x_*) layer was annealed at 200 °C, the efficiency was recovered to 17.1%, which was normal value typically observed in conventional structure PBDB‐T‐2F:Y6 solar cells. However, without any annealing process, the solar cell with aq‐MoO*_x_* exhibited comparable performance of 17.0%. In addition, the solar cell with annealing‐free aq‐MoO*_x_* exhibited better performance and stability without high‐temperature annealing compared to the solar cells with PEDOT:PSS. A similar trend was observed in a fullerene system solar cell based on a blend of poly[4,8‐bis(5‐(2‐ethylhexyl)thiophen‐2‐yl)benzo[1,2‐b:4,5‐b′]dithiophene‐alt‐3‐fluorothieno[3,4‐b]thiophene‐2‐carboxylate] (PTB7‐Th) and [6,6]‐phenyl‐C71‐butyric acid methyl ester (PC_71_BM). The PTB7‐Th:PC_71_BM solar cell with non‐annealed an‐MoO*_x_* showed very low efficiency of 1.37%, while the solar cell with annealing‐free aq‐MoO*_x_* exhibited a higher efficiency and better stability without high‐temperature annealing compared to the solar cells with PEDOT:PSS.

## 2 Results and Discussion

**Figure 1:**
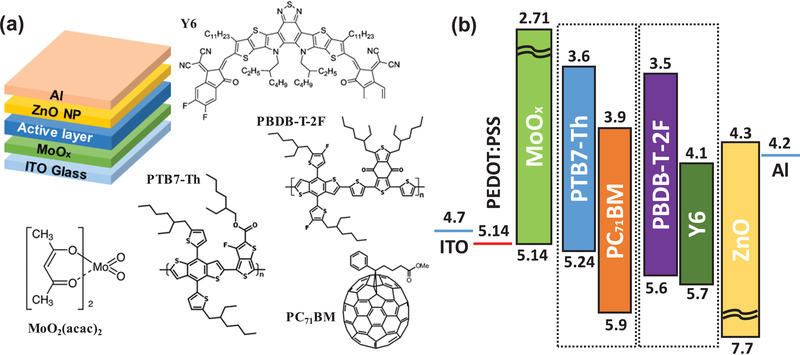
a) Schematic diagrams of the PSCs with a conventional structure as well as the chemical structure of the materials and b) the energy level diagram of the PSC with a conventional structure.

**Figure** **1**a shows the schematic diagrams of the PSCs with a conventional structure and the chemical structures of the materials used in this study. Molybdenyl acetylacetonate [MoO_2_(acac)_2_] was used as the starting material for preparing the an‐MoO*_x_* and aq‐MoO*_x_* solutions. The details regarding the fabrication of the an‐MoO*_x_* and aq‐MoO*_x_* solutions are described in the Experimental Section. Figure 1b shows the energy level diagram of the PSC with the conventional structure. To investigate the effect of aq‐MoO*_x_* on non‐fullerene solar cells, a combination of PBDB‐T‐2F and non‐fullerene acceptor Y6,^[^ ^30^, ^31^ ^]^ and a combination of poly[1‐(5‐(4,8‐bis(5‐(2‐ethylhexyl)thiophen‐2‐yl)‐6‐methylbenzo[1,2‐b:4,5‐b′]dithiophen‐2‐yl)‐4‐hexylthiophen‐2‐yl)‐3‐(4‐hexyl‐5‐methylthiophen‐2‐yl)‐5‐octyl‐4H‐thieno[3,4‐c]pyrrole‐4,6(5H)‐dione] (PBDTTPD‐HT) and 2,2′‐((2Z,2′Z)‐((4,4,9,9‐tetrahexyl‐4,9‐dihydro‐s‐indaceno[1,2‐b:5,6‐b′]dithiophene‐2,7‐diyl)bis(methanylylidene))bis(3‐oxo‐2,3‐dihydro‐1H‐indene‐2,1‐diylidene))dimalononitrile (IDIC) were utilized as the donor and acceptor materials.^[^ ^32^ ^]^ For the fullerene‐based active layer, PTB7‐Th and PC_71_BM were used as the photoactive materials. For the electrons transport layer, solution‐processable ZnO nanoparticles (nanoparticle suspension) that can be processed at room temperature were used.

**Figure 2:**
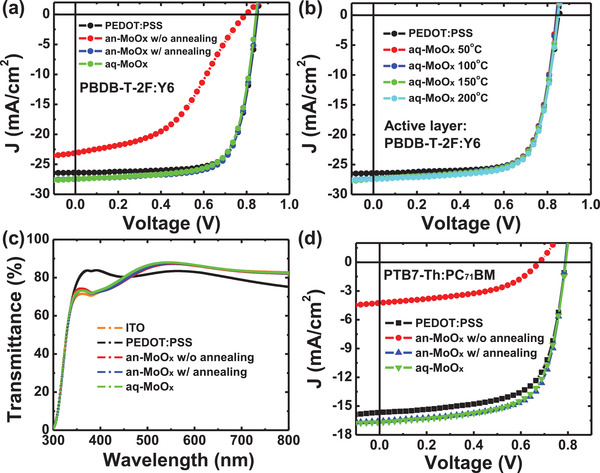
a) *J*–*V* characteristics of the PBDB‐T‐2F:Y6 solar cells, b) *J*–*V* characteristics of the PBDB‐T‐2F:Y6 solar cells with aq‐MoO*_x_* annealed at various temperatures, c) transmittance spectra of PEDOT:PSS, an‐MoO*_x_*, and aq‐MoO*_x_*, and d) *J*–*V* characteristics of the fullerene‐based solar cell based on PTB7‐Th:PC_71_BM.

**Figure** **2**a,d shows the current density (*J*) versus voltage (*V*) characteristics of the PBDB‐T‐2F:Y6 non‐fullerene solar cells (Figure 2a) and PTB7‐Th:PC_71_BM solar cells (Figure 2d) fabricated using aq‐MoO*_x_* or an‐MoO*_x_*, respectively. In addition, the *J–V* characteristics and photovoltaic parameters of the PBDTTPD‐HT:IDIC were shown in Figure S1 and Table S1, Supporting Information, respectively. The *J*–*V* results obtained from the device with the PEDOT:PSS HTL are presented for comparison. Note that the PEDOT:PSS film was annealed at 150 °C after spin‐coating. The external quantum efficiency (EQE) spectra of the PBDB‐T‐2F:Y6, PBDTTPD‐HT:IDIC non‐fullerene solar cells, and PTB7‐Th:PC_71_BM solar cells are presented in Figure S2, Supporting Information. The details related to the performance of PTB7‐Th:PC_71_BM and PBDB‐T‐2F:Y6 solar cells are listed in **Table** **1**. The statistics of photovoltaic parameters of the conventional polymer solar cell based on PTB7‐Th:PC_71_BM, PBDTTPD‐HT:IDIC, and PBDB‐T‐2F:Y6 were shown in Figures S3–S5, Supporting Information, respectively. The PBDB‐T‐2F:Y6 solar cell with the non‐annealed an‐MoO*_x_* HTL showed a very low power conversion efficiency of 7.7% with a relatively low short‐circuit current density (*J*_sc_) of 20.50 mA cm^−2^, an open‐circuit voltage (*V*_oc_) of 0.826 V, and a fill factor (FF) of 45.2%. These results are inevitable consequences of the general non‐annealed MoO*_x_* HTL prepared by the anhydrous sol–gel method because there was no chance to remove the ligands from the MoO*_x_* HTL without high‐temperature annealing. In fact, if the MoO*_x_* HTL is prepared with high‐temperature annealing at 200 °C, the efficiency of the solar cell fabricated with the an‐MoO*_x_* HTL recovered to a normal level of 17.1% with a *J*_sc_ of 27.43 mA cm^−2^, a *V*_oc_ of 0.845 V, and an FF of 73.8%. However, the solar cell with aq‐MoO*_x_* prepared by the aqueous sol–gel method showed almost comparable performance to the solar cell with the annealed an‐MoO*_x_* HTL, even without high‐temperature annealing. Note that the aq‐MoO*_x_* HTL was kept at 50 °C for 5–10 min to dry the surface because a hydrophobic organic active layer was not deposited well on top of the aq‐MoO*_x_* HTL without removing surface moisture. The solar cell with aq‐MoO*_x_* yields a PCE of 17.0% with a *J*_sc_ of 27.53 mA cm^−2^, a *V*_oc_ of 0.843 V, and an FF of 73.1%. The solar cell with aq‐MoO*_x_* exhibited better performance compared to the solar cell with the PEDOT:PSS HTL. Moreover, the performance of PBDB‐T‐2F:Y6 solar cells with the aq‐MoO*_x_* HTL did not change even after annealing at high temperatures of up to 200 °C, as shown in Figure 2b (Table S2, Supporting Information). The optical properties of these three MoO*_x_* HTLs did not differ significantly from each other, as shown in Figure 2c. However, the MoO*_x_* HTL exhibited a slightly higher transmittance in the visible region compared to PEDOT:PSS.

**Table 1:** Summarized photovoltaic parameters of the conventional polymer solar cell based on PTB7‐Th:PC_71_BM and non‐fullerene solar cell based on PBDB‐T‐2F:Y6

Active layer	Description	JV *J*_sc_ [mA cm^−2^]	EQE *J*_sc_ [mA cm^−2^]	*V*_oc_ [V]	FF [%]	Best PCE [%]
PBDB‐T‐2F and Y6	PEDOT:PSS	26.50	26.14 (98.6%)	0.854	0.735	16.6
	Anhydrous MoO*_x_*	20.50	20.02 (97.6%)	0.826	0.452	7.7
	Anhydrous MoO*_x_* (200 °C)	27.43	26.87 (97.9%)	0.845	0.738	17.1
	Aqueous MoO*_x_*	27.53	26.91 (97.7%)	0.843	0.731	17.0
PTB7‐Th and PC_71_BM	PEDOT:PSS	15.67	14.88 (94.9%)	0.791	0.673	8.3
	Anhydrous MoO*_x_*	4.25	4.12 (96.9%)	0.683	0.479	1.4
	Anhydrous MoO*_x_* (200 °C)	16.60	16.35 (98.5%)	0.791	0.682	9.0
	Aqueous MoO*_x_*	16.69	16.20 (97.1%)	0.790	0.671	8.9

A similar trend was observed in the fullerene‐based solar cell using PTB7‐Th:PC_71_BM (see Figure 2d). The solar cell with the non‐annealed an‐MoO*_x_* HTL showed a PCE of just 1.4% with a poor FF (47.9%) and a very low *J*_sc_ (4.25 mA cm^−2^) together with a larger series resistance. When the an‐MoO*_x_* HTL was annealed at 200 °C, however, the efficiency was recovered to 9.0% (see Figure S6 and Table S3, Supporting Information). With the aq‐MoO*_x_* HTL, the PTB7‐Th:PC_71_BM solar cell exhibited a comparable efficiency of 8.9% even without any annealing process. The *J*–*V* curves and photovoltaic parameters of polymer solar cells based on PTB7‐Th:PC_71_BM with aq‐MoO*_x_* with various H_2_O concentrations were shown in Figure S7 and Table S4, Supporting Information.

**Figure 3:**
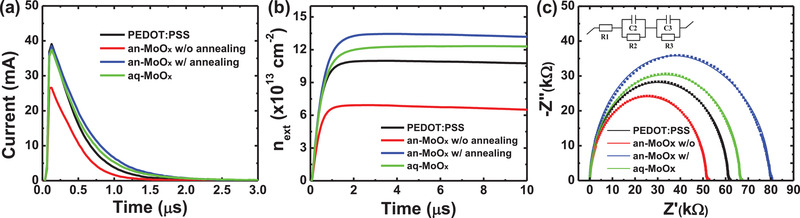
a) Charge extraction (CE) current transient under 1 sun illumination and *V*_oc_ conditions, b) calculated charge extraction density, and c) Nyquist plot of the impedance spectroscopy measurements of the polymer solar cells with various MoO*_x_* HTLs under dark conditions.

In the evaluation of the solar cell performance, the aq‐MoO*_x_* HTL clearly performed better than the PEDOT:PSS HTL. **Figure** **3**a shows the charge extraction (CE) current, which was measured under 1‐sun illumination and *V*_oc_ conditions. From this CE current, the CE density was deduced, as shown in Figure 3b. The CE density results were consistent with the *J*–*V* characteristics. In the case of the device with the PEDOT:PSS HTL, the calculated CE density was 1.09 × 10^14^ cm^−2^. The device with the non‐annealed an‐MoO*_x_* HTL showed a one order of magnitude lower CE density of 6.93 × 10^13^ cm^−2^ than the device with the PEDOT:PSS HTL. However, similar to the *J*–*V* results, the CE density of the device with the an‐MoO*_x_* HTL significantly increased with high temperature annealing at 200 °C. In the case of the device with the aq‐MoO*_x_* HTL, although the calculated CE density value was slightly lower than that of the device with the annealed an‐MoO*_x_* HTL (1.35 × 10^14^ cm^−2^), it clearly showed a better CE density value (1.23 × 10^14^ cm^−2^) than that of the device with the PEDOT:PSS HTL.

The improved electrical properties of the aq‐MoO*_x_* HTL were also observed in the impedance spectroscopy (IS) study. The Nyquist plots of the IS results measured under dark conditions are shown in Figure 3c. All of the devices exhibited one semicircle without a transmission line (TL), which indicates that all devices undergo strong recombination.^[^ ^33^ ^]^ In general, this feature can be interpreted using the Gerischer impedance model, where the square root of the product of the recombination resistance (*R*_rec_) and transportation resistance (*R*_tr_) can be described as the Gerischer resistance (*R*_G_).^[^ ^34^, ^35^ ^]^ The device with the annealed an‐MoO*_x_* HTL or aq‐MoO*_x_* HTL exhibited a higher *R*_G_ (or *R*_rec_, which is inversely proportional to the recombination rate) than the device with the non‐annealed an‐MoO*_x_* HTL or PEDOT:PSS. Since all devices have the same structure except for the MoO*_x_* layer, the difference of *R*_G_ definitely resulted from the charge transportation at the MoO*_x_* layer and charge recombination at the interface. A higher *R*_G_ corresponds to a lower recombination probability, which indicates better charge transport ability without recombination. Therefore, the IS results shown in Figure 3c clearly indicate that the charge transport properties of the aq‐MoO*_x_* HTL was better than that of the PEDOT:PSS HTL.

**Figure 4:**
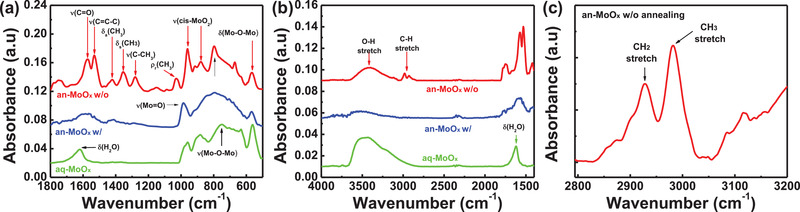
Fourier transform infra‐red (FTIR) spectroscopy of MoO*_x_* HTLs: a) fingerprint region, b) function group region, and c) CH_2_ and CH_3_ stretch mode vibration peaks of non‐annealed an‐MoO*_x_* films.

Normally, in metal oxide layers prepared by an anhydrous sol–gel method, high‐temperature curing is conducted to remove ligands, which provide solubilizing power in metal oxide nanoparticles. To explore the chemical structure differences between the an‐MoO*_x_* HTL and aq‐MoO*_x_* HTL, Fourier transform infra‐red (FTIR) spectroscopy was performed, as shown in **Figure** **4**. The non‐annealed an‐MoO*_x_* layer showed clear evidence of the characteristic peaks of acetylacetonate in the fingerprint region including a C=C stretch at 1573 cm^−1^, C=O stretch at 1530 cm^−1^, C=O stretch + C—H bending at 1422 cm^−1^, CH_3_ symmetrical bending at 1354 cm^−1^, C=C stretch + C—CH_3_ stretch at 1280 cm^−1^, and CH_3_ rocking at 1029 cm^−1^.^[^ ^36^, ^37^, ^38^ ^]^ In addition, the non‐annealed an‐MoO*_x_* layer also exhibited a broad peak at 3300 cm^−1^, which is attributed to the O—H stretch vibration of ethanol, and two C—H stretch peaks near 2980 cm^−1^, which are attributed to acetylacetonate (Figure 4b).^[^ ^39^ ^]^ However, in the an‐MoO*_x_* layer cured at 200 °C, all carbon related peaks disappeared, which indicates that acetylacetonate ligands were successfully removed by the curing process. The chemical structure change of the an‐MoO*_x_* layer as a function of the curing temperature is presented in Figure S8, Supporting Information. The removal of acetylacetonate peaks from the an‐MoO*_x_* layer was only completed at 200 °C, which is in good agreement with previous literatures.^[^ ^28^, ^29^ ^]^ For the aq‐MoO*_x_* layer, however, the acetylacetonate peaks almost vanished in the FTIR spectra even without a curing process. The aq‐MoO*_x_* layer showed a bending and stretching vibrations of H_2_O at 1620 and 3300 cm^−1^, respectively. Therefore, it can be concluded that all of the acetylacetonate ligands were already removed from the solution state by the hydrolysis effect resulting from the added water. The FTIR spectra of the aq‐MoO*_x_* layer as a function of the curing temperature is shown in Figure S9, Supporting Information. With increasing curing temperature, only water peaks were removed. However, the presence of water does not seem to significantly influence the performance of the solar cells (Figure 2c).

The non‐annealed an‐MoO*_x_* exhibited peaks at 960, 880, 800, and 564 cm^−1^. The peak at 800 cm^−1^ was characterized as the C—H out‐of‐plane bending mode originating from acetylacetonate. The doublet peak at 960 and 880 cm^−1^ was characterized as the stretching mode of cis‐MoO_2_, O=Mo=O, while the peak at 564 cm^−1^ was identified as the bending mode of Mo—O—Mo.^[^ ^40^, ^41^ ^]^ The peak in the range of 850–600 cm^−1^ was identified as the stretching mode of Mo—O—Mo.^[^ ^28^, ^36^, ^42^ ^]^ In addition, only non‐annealed an‐MoO*_x_* exhibited the CH_2_ stretching mode at 2927 cm^−1^ (Figure 4c), which indicates the possibility of interaction between ethanol and MoO_2_(acac)_2_. Based on all of the FTIR results, it can be concluded that the non‐annealed an‐MoO*_x_* film could be described as Mo_2_O_5_(acac)_2_(EtOH)_2_.^[^ ^40^ ^]^ In the an‐MoO*_x_* film annealed at high temperature, the C—H out‐of‐plane bending mode peak at 800 cm^−1^ disappeared due to the decomposition of the acetylacetonate ligand. Furthermore, the cis‐MoO_2_ peak at 880 cm^−1^ disappeared and the peak at 960 cm^−1^ was slightly shifted to 985 cm^−1^, which is attributed to Mo=O stretching. In addition, the CH_2_ stretching mode peak also disappeared. All of these phenomena observed after high‐temperature annealing can be interpreted as the process of conversion from the cis‐MoO_2_ form with ligands to a form close to MoO_3_.^[^ ^43^ ^]^ For the aq‐MoO*_x_*, the overall features of the spectrum in the 1100–500 cm^−1^ region were similar to annealed an‐MoO*_x_*. No C—H related peaks were observed. The board peak at 880 cm^−1^ in aq‐MoO*_x_* was interpreted as a MoO_3_(H_2_O)*_x_* stretching mode. The IR spectrum of aq‐MoO*_x_* showed a similar feature of molybdenum trioxide hydrates.^[^ ^40^, ^43^, ^44^ ^]^ Although the intermediate hydrate Mo_2_O_5_(acac)_2_(H_2_O)_2_ state was not observed in our study, the overall FTIR results clearly indicate that MoO_2_(acac)_2_ was successfully converted to (MoO_3_)*_n_*(H_2_O)_2_ *_n_*_+1_ without high temperature curing due to hydrolysis with added water.

**Figure 5:**
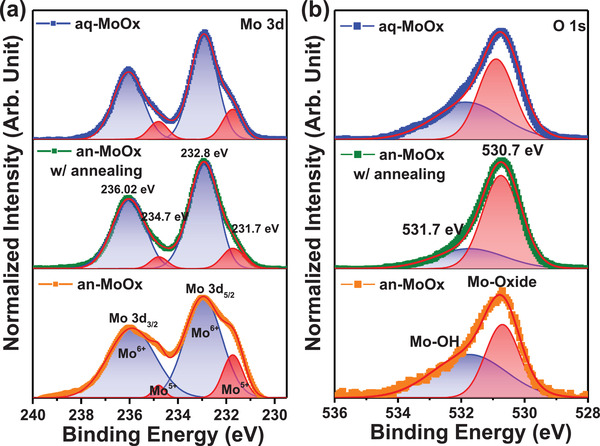
XPS spectra of a) Mo 3d and b) O 1s core level peaks obtained from the MoO*_x_* layers.

X‐ray photoelectron spectroscopy (XPS) analysis was carried out to more clearly elucidate the state of the Mo atoms. **Figure** **5**a,b shows the XPS spectra of the Mo 3d and O 1s peaks, respectively. For the Mo 3d peaks, by using the Gaussian fitting method, the two Mo 3d peaks were divided into two doublets originating from the spin‐orbital splitting. The major doublet (higher intensity) at 232.8 and 236.02 eV belonged to the Mo^6+^ oxidation state, while the minor doublet (lower intensity) at 231.7 and 234.5 eV belonged to the Mo^5+^ oxidation state.^[^ ^29^ ^]^ The atomic concentration ratio of Mo^6+^/Mo^5+^ was determined from the proportion of the integrated peak area of each component. The Mo^6+^/Mo^5+^ ratios were 3.86 and 4.59 for an‐MoO*_x_* without annealing and aq‐MoO*_x_*, respectively. For an‐MoO*_x_* with annealing, the Mo^6+^/Mo^5+^ ratio was 5.13. The O 1s peak was analyzed as two peaks by Gaussian fitting. The lower binding energy peak (oxygen‐LBE) at 530.7 eV is assigned to bonding with the Mo atom, while the higher binding energy peak (oxygen‐HBE) at 531.7 eV is attributed to the oxygen associated with the acetylacetonate ligand.^[^ ^28^ ^]^ The oxygen‐HBE/oxygen‐LBE ratios were 1,28, 0.41, and 0.94 for an‐MoO*_x_* without annealing, an‐MoO*_x_* with annealing, and aq‐MoO*_x_*, respectively.

**Figure 6:**
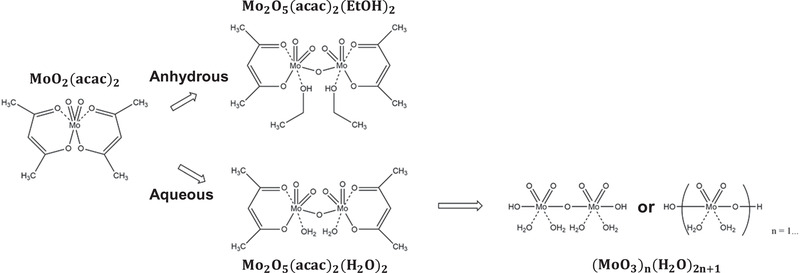
Schematic illustration of the expected chemical structures of the an‐MoO*_x_* and aq‐MoO*_x_* precursor solutions.

Among the three MoO_x_ HTLs, the annealed an‐MoO*_x_* showed a higher Mo^6+^/Mo^5+^ ratio and lower oxygen‐HBE/oxygen‐LBE ratio compared to an‐MoO*_x_* without annealing, which means that the Mo^6+^ fraction and oxygen‐LBE fraction were enhanced after high‐temperature annealing. These results clearly indicate that Mo^5+^ was further oxidized to Mo^6+^ due to decomposition of the acetylacetonate ligand.^[^ ^45^ ^]^ A similar trend was observed in the comparison between annealed an‐MoO*_x_* and aq‐MoO*_x_*. The aq‐MoO*_x_* showed a higher Mo^6+^/Mo^5+^ ratio and lower oxygen‐HBE/oxygen‐LBE ratio compared to an‐MoO*_x_* without annealing. Therefore, we can conclude that water removed the acetylacetonate ligand from MoO_2_(acac)_2_ and oxidized Mo atoms to the highest oxidation state of Mo^6+^. Interestingly, the Mo:O stoichiometry of all MoO*_x_* layers was below than 1:3, which indicates that all MoO*_x_* layers were sub‐stoichiometric. This is the reason why we used MoO*_x_* instead of MoO_3_. The Mo:O stoichiometry is listed in Table S5, Supporting Information. Consequently, we clearly confirmed that the acetylacetonate ligand can be removed not only by high‐temperature curing but also by the hydrolysis process of water. Based on the results of the FTIR and XPS studies, the expected chemical structures of the an‐MoO*_x_* and aq‐MoO*_x_* precursor solutions are illustrated in **Figure** **6**.

**Figure 7:**
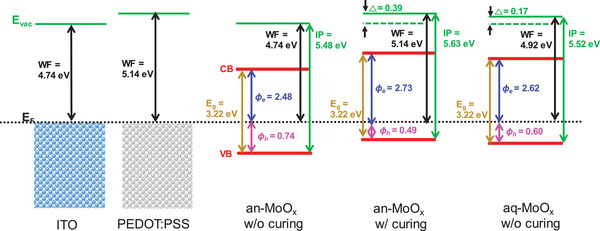
Energy band diagrams of aq‐MoO*_x_* and an‐MoO*_x_* with and without annealing layers obtained from UPS measurements.

To investigate the band structure difference between an‐MoO*_x_* and aq‐MoO*_x_*, ultraviolet photoelectron spectroscopy (UPS) analysis was conducted, as shown in Figure S10, Supporting Information. The summary of the UPS analysis is illustrated in **Figure** **7** as band diagrams. The Fermi energy (*E*_F_) was calculated from the ITO surface and all other spectra are plotted with respect to this value. The calculated *E*_F_ values were 4.74 and 5.14 eV for ITO and PEDOT:PSS, respectively. The vacuum levels (VLs) were determined by linear extrapolation of the secondary electron cutoffs on the high binding energy side. The valence band (VB) maximum was extracted from the onset on the low binding energy side. The relative position of the VB maximum level was determined by comparing the shift of the onset to the *E*_F_ of ITO. The conduction band (CB) minimum level was estimated using the extracted VB maximum and the measured optical gaps extracted from the UV–vis absorption spectra (Figure S11 and Table S6, Supporting Information). All MoO*_x_* layers exhibited an optical band gap of 3.22 eV, which are in agreement with the literature data.^[^ ^46^ ^]^ The workfunction (WF) of the non‐annealed an‐MoO*_x_* HTL was 4.74 eV, which is comparable to that of the ITO substrate. The WF of the an‐MoO*_x_* HTL with annealing was enlarged due to the VL shift of 0.39 eV. In the case of aq‐MoO*_x_*, the measured WF was 4.92 eV. A similar trend was observed for the calculated hole injection barrier (*φ*_h_). The deduced *φ*_h_ values were 0.74, 0.49, and 0.60 eV for the non‐annealed an‐MoO*_x_* HTL, an‐MoO*_x_* HTL with annealing, and aq‐MoO*_x_*, respectively. The an‐MoO*_x_* HTL with annealing showed more favorable energy level alignment for hole transport. The *φ*_h_ value of aq‐MoO*_x_* was slightly higher than that of the annealed an‐MoO*_x_* HTL. This energy level difference played an important role in the performance of the polymer solar cell. All of the solar cell results were consistent with this *φ*_h_ difference. Of course, the energy level alignment of aq‐MoO*_x_* was not the best, but it is worth noting that a significant improvement was achieved without high temperature curing. To get more insight into the charge transport property at the interface between HTLs and active layer, we have measured hole mobilities using hole‐only devices with structure ITO/HTLs/active layer/evaporated MoO*_x_*/Ag. The active layer thickness was 150 nm and the thickness of HTLs was shown in Table S7, Supporting Information. The active layer permittivity was 3.5, as previously stated in literature.^[^ ^47^ ^]^ The hole mobilities of various hole transport layers (HTLs)/active layer were determined by the steady‐state space‐charge‐limited current (SCLC) method. The SCLC region and hole mobility of various HTLs/active layer were shown in Figure S12 and Table S8, Supporting Information, respectively. The hole mobility of PEDOT:PSS/PBDB‐T‐2F:Y6 was similar to other studies reported in literature.^[^ ^30^ ^]^ The distinguishable difference in the hole mobility of an‐MoO*_x_* with and without annealing showed a clear effect of high temperature annealing in the removal of the organic ligand. Furthermore, the hole mobility of aq‐MoO*_x_* was higher than that of PEDOT:PSS. Overall, the hole mobility data in Table S8, Supporting Information, is consistent with the *J*–*V* curves of polymer solar cells.

In addition to the energy level alignment, morphology changes can also influence the charge transport properties. However, a significant surface morphology change was not detected in the atomic force microscope (AFM) measurements (Figure S13, Supporting Information). Furthermore, the morphology of PBDB‐T‐2F:Y6 casted on various hole transport layers was also examined (Figure S14, Supporting Information). As shown in Figure S14, Supporting Information, all AFM images showed similar fibril network morphology. Therefore, we concluded that the difference of the performance of the solar cell was not due to a morphology change at the interface between the MoO*_x_* HTL and active layer.

**Figure 8:**
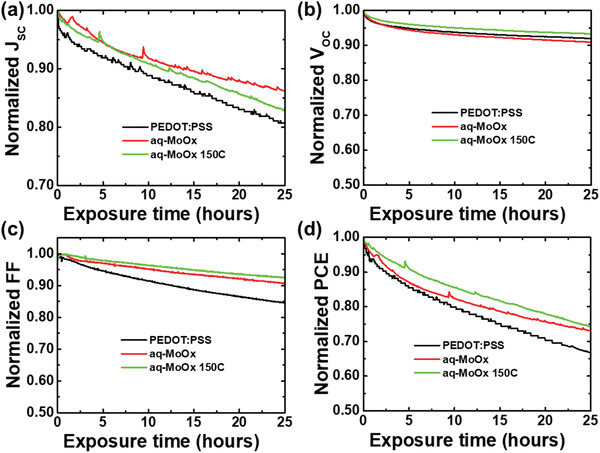
Changes of a) *J*_sc_, b) *V*_oc_, c) FF, and d) PCE of non‐fullerene solar cell based on PBDB‐T‐2F:Y6 during continuous operation in air without any encapsulation.

Since the aq‐MoO*_x_* HTL does not require an annealing process, the water added during the sol–gel process remains in the film (Figure 4). Of course, it is expected to be in a weakly bonded form, but it is doubtful that the remaining water will affect the stability of the device. To confirm this point, we evaluated the operation stability for the PBDB‐T‐2F:Y6 non‐fullerene solar cells. This stability test was conducted in air without any encapsulation. **Figure** **8** shows the changes of *J*_sc_, *V*_oc_, FF, and PCE measured continuously over 25 h with various HTLs as PEDOT:PSS, aq‐MoO*_x_* without annealing, and aq‐MoO*_x_* annealed at 150 °C. Because of well‐known weak stability of non‐fullerene solar cells based on Y6, both solar cell with the PEDOT:PSS and solar cell with MoO*_x_* HTL showed continuous decreases of PCE from the beginning.^[^ ^48^ ^]^ However, after 25 h of operation, the solar cell with aq‐MoO*_x_* showed better condition compared to the solar cell with PEDOT:PSS.

Storage stability performed for PTB7‐Th:PC_71_BM solar cells also showed similar trend (Figure S15, Supporting Information). The PTB7‐Th:PC_71_BM solar cell with the PEDOT:PSS HTL showed continuous decreases of *J*_sc_ and FF from the beginning. After 1000 h, the PCE significantly decreased from 8.43% to 4.9%. In the case of the solar cell with the aq‐MoO*_x_* HTL, however, all of the solar cell parameters were well maintained compared to the solar cell with PEDOT:PSS. The PCE decreased by only 9% from 8.81% to 8.01%. Therefore, it can be concluded that the aq‐MoO*_x_* HTL does not significantly affect the stability even if it contains water in the film.^[^ ^49^ ^]^

**Figure 9:**
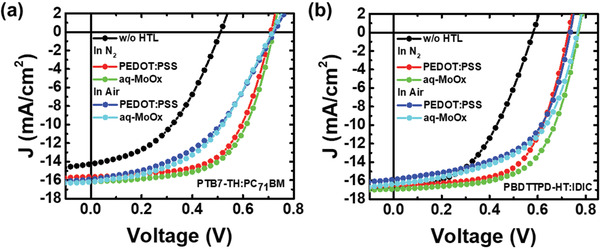
a) *J*–*V* characteristics of the inverted polymer solar cells based on PTB7‐Th:PC_71_BM and the b) *J*–*V* characteristics of polymer solar cell based on PBDTTPD‐HT:IDIC. The hole transport layers were deposited on active layer in N_2_ or air. To obtain better adsorption with an organic active layer, 2 mg mL^−1^ of Triton X‐100 was added into the PEDOT:PSS and aq‐MoO*_x_* solution.

The most notable advantage of annealing‐free aq‐MoO*_x_* is that it allows the fabrication of an inverted solar cell employing only a solution process. Some organic photoactive materials become significantly degraded with high temperature annealing including PTB7‐Th:PC_71_BM and PBDTTPD‐HT:IDIC combinations. The PTB7‐Th and PC_71_BM active layer allows only a maximum temperature of 80 °C. Thus, in most cases, PTB7‐Th and PC_71_BM solar cells were fabricated with room temperature drying by keeping them in a glove box for 2–3 h. The PBDTTPD‐HT:IDIC active layer is similar as it also allows only a maximum of 100 °C annealing after active layer deposition. Therefore, the fabrication of inverted solar cells was not possible using general solution‐processable HTL materials, which require high‐temperature curing over 150 °C. In most cases, a vacuum deposition method was utilized for the HTL construction. The use of PEDOT:PSS was also limited in the fabrication of inverted solar cells because PEDOT:PSS also needed to be dried at a temperature of at least 100 °C. However, with our aq‐MoO*_x_* HTL, active layer degradation by high temperature annealing is no longer a serious problem. **Figure** **9** shows the *J*–*V* characteristics of the PTB7‐Th:PC_71_BM and PBDTTPD‐HT:IDIC solar cell fabricated with an inverted structure. The HTLs were deposited on active layer in N_2_ or in air. In both PTB7‐Th:PC_71_BM and PBDTTPD‐HT:IDIC active layer combinations, the solar cells with aq‐MoO*_x_* showed slightly better performance compared to the solar cell with the PEDOT:PSS HTL. Somewhat lower performance compared to the solar cell with the conventional structure was obtained because of the adsorption property. Since aq‐MoO*_x_* is based on a hydrophilic solvent, it is somewhat difficult to deposit it on a hydrophobic organic photoactive layer by spin‐coating. Therefore, to enhance the adsorption property, we added 2 mg mL^−1^ of Triton X‐100 into the PEDOT:PSS and aq‐MoO*_x_* solution.^[^ ^50^, ^51^ ^]^ This performance decrease was most likely caused by adding Triton X‐100.

## 3 Conclusion

In summary, we developed annealing‐free solution‐processable aqueous MoO*_x_* and applied it in a BHJ polymer solar cell based on a blend of PBDB‐T‐2F:Y6 and PTB7‐Th:PC_71_BM. In general, the charge transport layer based on moly oxides prepared by an anhydrous sol–gel method normally requires high‐temperature annealing in air to achieve desired quality. As a result, the non‐fullerene solar cell with the general an‐MoO*_x_* HTL prepared by the sol–gel method showed a poor efficiency of 7.7% without annealing. This poor efficiency was recovered to the normal value typically observed in PBDB‐T‐2F:Y6 solar cells when this an‐MoO*_x_* layer was annealed at 200 °C. However, the solar cell with aq‐MoO*_x_* prepared by the aqueous sol–gel method showed almost comparable performance to the solar cell with the annealed an‐MoO*_x_* HTL, even without high‐temperature annealing. Moreover, the solar cell with aq‐MoO*_x_* exhibited a higher efficiency and better stability without high‐temperature annealing compared to the solar cells with PEDOT:PSS. A similar trend was observed in the fullerene‐based solar cell based on PTB7‐Th:PC_71_BM. In addition, annealing‐free aq‐MoO*_x_* allowed the successful fabrication of inverted PTB7‐Th:PC_71_BM solar cell, which was impossible to fabricate using a general anhydrous MoO*_x_* HTL which requires a high‐temperature annealing process. Although high‐temperature annealing is not a difficult process in laboratory scale production, it is definitely not a simple process to apply in roll‐to‐roll processes because of the inevitable long cooling step that follows. Thus, our aqueous method based on the hydrolysis effect can be an essential technique for large‐scale roll‐to‐roll mass production of flexible devices.

## 4 Experimental Section

##### Preparation of the MoO*_x_* Solution

Molybdenum dioxydiacetylacetonate (MoO_2_(acac)_2_ 99%) was purchased from Sigma‐Aldrich and used as the precursor material for the aqueous and anhydrous MoO*_x_* solutions. For the anhydrous solution, MoO_2_(acac)_2_ (12 mg mL^−1^) was diluted in 10 mL of anhydrous ethanol 99.9% and stirred for 1 h to obtain a clear and colorless solution. In the case of the aqueous solution, MoO_2_(acac)_2_ (12 mg mL^−1^) was diluted in a mixture of 1 mL anhydrous of ethanol 99.9% (Sigma‐Aldrich) and 9 mL of deionized water. Ethanol was added to improve the solubility of MoO_2_(acac)_2_ and hydrophilicity of aq‐MoO*_x_* solution. The aqueous solution was stirred for 1 h to obtain a light‐blue solution. Both aqueous and anhydrous solutions were kept at room temperature for aging over 10 days, which were needed for complete hydrolysis process of MoO*_x_* solution, to obtain ready to use dark‐blue color solutions. To prepare the MoO*_x_* solution used for the inverted polymer solar cell, Triton X‐100 (Sigma‐Aldrich) was mixed with the MoO*_x_* aqueous solution at a ratio of 2:4 (mg:mL), which was denoted as ratio 0.5. The Triton X‐100:MoO*_x_* solution was ultrasonicated for 2 h and kept at room temperature.

##### Device Fabrication

The conventional BHJ polymer solar cells were fabricated using an ITO/MoO*_x_*/PTB7‐Th:PC_71_BM/ZnO/Al structure. The patterned indium‐tin oxide (ITO) substrates were subjected to ultrasonication in deionized water, acetone, and isopropyl alcohol, respectively. They were then dried in an oven at 100 °C for several hours. Once dried, the substrates were treated with UV for 60 min to increase the hydrophilicity. The MoO*_x_* film was coated on the cleaned ITO substrates by spin coating the aqueous MoO*_x_* solution or anhydrous MoO*_x_* solution at 5000 rpm followed by annealing at 50 or 200 °C for 10 min in air. Then, the MoO*_x_* film was cooled to room temperature and then placed into an N_2_‐filled glovebox to make the solar cell active layer. The PTB7‐Th:PC_71_BM (1:1.5 by weight) blend solution was prepared by dissolving in chlorobenzene with 3% DIO as an additive to achieve better phase separation. The PBDTTPD‐HT:IDIC (1:1.5 by weight) blend solution was prepared by dissolving in chloroform with 0.6% DIO additive. The PBDB‐T‐2F:Y6 (1:1.2 by weight) blend solution was prepared by dissolving in chloroform with 0.5% CN additive. The solution was stirred for 1 day before using. After passing through a 0.45 µm PTFE syringe filter, the blend solution was spin‐cast onto the MoO*_x_* layer with a spin speed ranging from 900 to 1100 rpm to create an approximately 100 nm thick active layer of PTB7‐Th:PC_71_BM. In case of PBDTTPD‐HT:IDIC and PBDB‐T‐2F:Y6, a spin speed ranging from 3000 to 4000 rpm was used. A ZnO nanoparticle solution (2.5 wt% in IPA) diluted in IPA at a ratio of 1:5 was spin‐coated on top of the active layer at 5000 rpm. The devices were kept inside the glovebox for 1 h for drying at room temperature. Then, the devices were placed in the evaporation chamber to make 100 nm of Al under a high vacuum pressure of less than 10^−6^ Torr. The active surface area of the device, as defined by a metal shadow mask, was 0.13 cm^2^. For the reference device, PEDOT:PSS (CLEVIOS PH1000), which was substituted for the MoO*_x_* layer, was spin‐coated on top of ITO substrate at 5000 rpm and annealed at 150 °C in air.

The inverted BHJ polymer solar cells were fabricated using an ITO/ZnO/PTB7‐Th:PC_71_BM/MoO*_x_*/Ag structure. A ZnO nanoparticle solution (2.5 wt% in IPA) diluted in IPA at a ratio of 1:1 was spin‐coated on top of the ITO substrate at 5000 rpm and annealed at 85 °C for 10 min in air. Then, the ZnO film was cooled to room temperature and then placed in an N_2_‐filled glovebox to make the solar cell active layer. The PTB7‐Th:PC_71_BM blend solution was spin‐cast onto the ZnO layer at 1000 rpm. A Triton X‐100:MoO*_x_* aqueous solution was spin‐coated on top of the active layer at 3000 rpm and the devices were dried inside the glovebox for 1 h without using any post‐treatment annealing temperature. For the reference device, modified PEDOT:PSS (M‐PEDOT) was spin‐coated on top of the active layer at 5000 rpm and annealed at 80°C for 10 min. Then, the devices were placed in the evaporation chamber to make 100 nm of Ag under a high vacuum pressure of less than 10^−6^ Torr and the active surface area of the device was 0.13 cm^2^. The non‐fullerene polymer solar cell was fabricated using the same procedure described above, except that PBDTTPD‐HT:IDIC was used as the polymer active layer.

##### Device Characteristics

The current density–voltage curves of the solar cell devices were obtained using a Keithley 2401 source measurement unit under AM1.5G simulated illumination (100 mW cm^−2^). A standard Si photodiode detector with a KG‐3 filter (Newport Co., Oriel) was used to calibrate the intensity of the simulated sunlight. All devices were measured under an inert environment using a solar simulator inside an N_2_‐filled glove box. The EQE of the optimized solar cell performance was determined by the IQE‐200B (Newport Co., Oriel). The absorption and transmittance spectra of MoO*_x_* were measured by using a UV–vis spectrophotometer (Varian, Carry 5000). The surface morphologies of the MoO*_x_* layers and Triton X‐100:MoO*_x_* layer were measured by using an atomic force microscope (AFM, Nanocute, SII NanoTechnology). UPS and XPS were carried out with an ESCALAB 250‐XI surface analysis system equipped with a He‐discharge lamp providing He‐I photons with an energy of 21.22 eV for UPS analysis and a monochromatic Al‐K*α* X‐ray gun with a photon energy of 1486.6 eV for the XPS investigation. The base vacuum pressure of the analysis system was ≈10^−7^ Torr. The Fermi edge was calibrated using a clean Au film and all spectra were plotted with respect to the determined Fermi level. All XPS measurements were calibrated with reference to the Au 4f_7/2_ core level (83.98 eV) of a freshly deposited Au film. All binding energies were normalized with the C 1s peak as an internal standard. CE and CELIV were conducted using the CE and CELIV analyzer functions of an organic semiconductor parameter test system (McScience T4000) under 1.0 sun at *V*_OC_ conditions. FTIR was conducted using a Bruker Invenio R spectrometer. Impedance spectroscopy (IS) was carried out by using an impedance analyzer (IVIUM tech, IviumStat) under dark conditions, which measured the optoelectronic frequency response in the frequency range of 1 MHz to 1 Hz. The thickness of various hole transport layers was determined by using Stylus Profilometry (Bruker Dektak XT). The hole mobilities of various hole transport layers (HTLs)/active layer were measured by the SCLC method. The structures of the hole‐only device were ITO/HTLs/PBDB‐T‐2F:Y6/MoO*_x_*/Ag. The hole mobility of HTLs/active layer was determined by Mott–Gurney law in the SCLC trap free regime, by using the slope of *J* ^0.5^ versus *V*.

## Conflict of Interest

The authors declare no conflict of interest.

## Supporting information

Supporting Information
