# Supplementary material for: 17% Non‐Fullerene Organic Solar Cells with Annealing‐Free Aqueous MoOx
Source: Adv Sci (Weinh). 2020 Sep 21;7(21):2002395. doi: 10.1002/advs.202002395 (PMC7610336; doi:10.1002/advs.202002395)
Supplement: Supplementary file 1 — Supporting Information [file ADVS-7-2002395-s001.pdf]

© 2020 Wiley-VCH GmbH

ADVANCED SCIENCE
Open Access

Supporting Information

for *Adv. Sci.,* DOI: 10.1002/advs.202002395

17% Non-Fullerene Organic Solar Cells with Annealing-Free Aqueous MoO_x

Hong Nhan Tran, Sujung Park, Febrian Tri Adhi Wibowo, Narra Vamsi Krishna, Ju Hwan Kang, Jung Hwa Seo, Huy Nguyen-Phu, Sung-Yeon Jang, and Shinuk Cho**

SUPPORTING INFORMATION

17% non-fullerene organic solar cells with annealing-free aqueous MoO_x

Hong Nhan Tran,[a] Sujung Park,[a] Febrian Tri Adhi Wibowo,[b] Narra Vamsi krishna,[b] Ju Hwan Kang,[c] Jung Hwa Seo,[c] Huy Nguyen-Phu,[d] Sung-Yeon Jang,[b,]* and Shinuk Cho[a,]*

[a]Department of Physics and EHSRC, University of Ulsan, Ulsan 44610, Republic of Korea
[b]School of Energy and Chemical Engineering, Ulsan National Institute of Science and Technology, Ulsan 44919, Republic of Korea
[c]Department of Materials Physics, Dong-A University, Busan 49315, Republic of Korea
[d]School of Chemical Engineering, University of Ulsan, Ulsan 44610, Republic of Korea

*Corresponding author e-mail: *sucho@ulsan.ac.kr* (S. Cho) and *syjang@unist.ac.kr* (S.-Y. Jang)

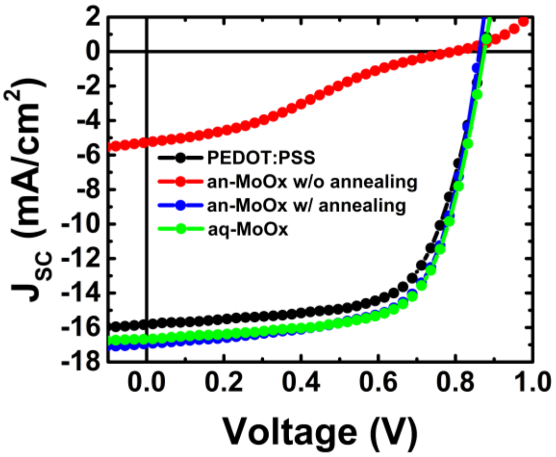

Figure S1. *J-V* characteristics of the conventional polymer solar cell based on PBDTTPD-HT:IDIC blend films with various hole transport layers.

Table S1 Photovoltaic performance of the conventional polymer solar cell based on PBDTTPD-HT:IDIC blend films with various hole transport layers.

Active Layer	Description	JV J_{sc} [mA/cm^2]	EQE J_{sc} [mA/cm^2]	V_{oc} [V]	FF [%]	Best PCE [%]
PBDTTPD-HT & IDIC	PEDOT:PSS	15.82	15.25 (96.4%)	0.87	0.66	9.08
	Anhydrous MoO_x	5.27	4.92 (93.4%)	0.80	0.30	1.26
	Anhydrous MoO_x (200 ^{o}C)	16.94	16.70 (98.6%)	0.86	0.66	9.62
	Aqueous MoO_x	16.68	16.43 (98.5%)	0.87	0.67	9.72

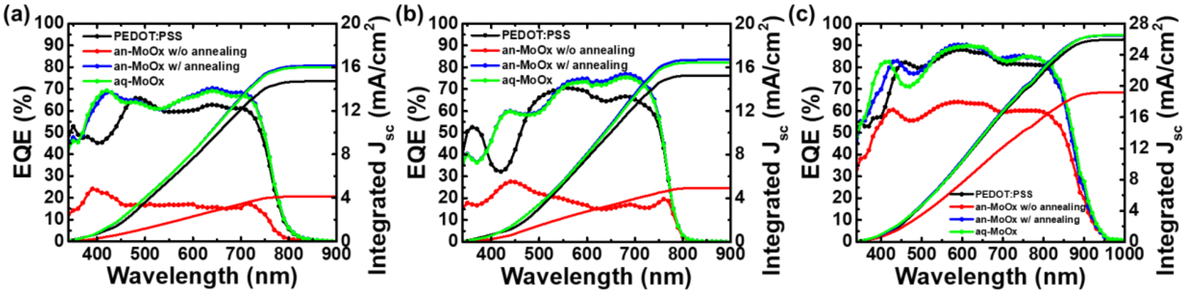

Figure S2. (a) EQE spectra of the solar cell based on PTB7-Th and $PC_{71}BM$. (b) EQE spectra of the solar cell based on PBDTTPD-HT and IDIC. (c) EQE spectra of the solar cell based on PBDB-T-2F and Y6.

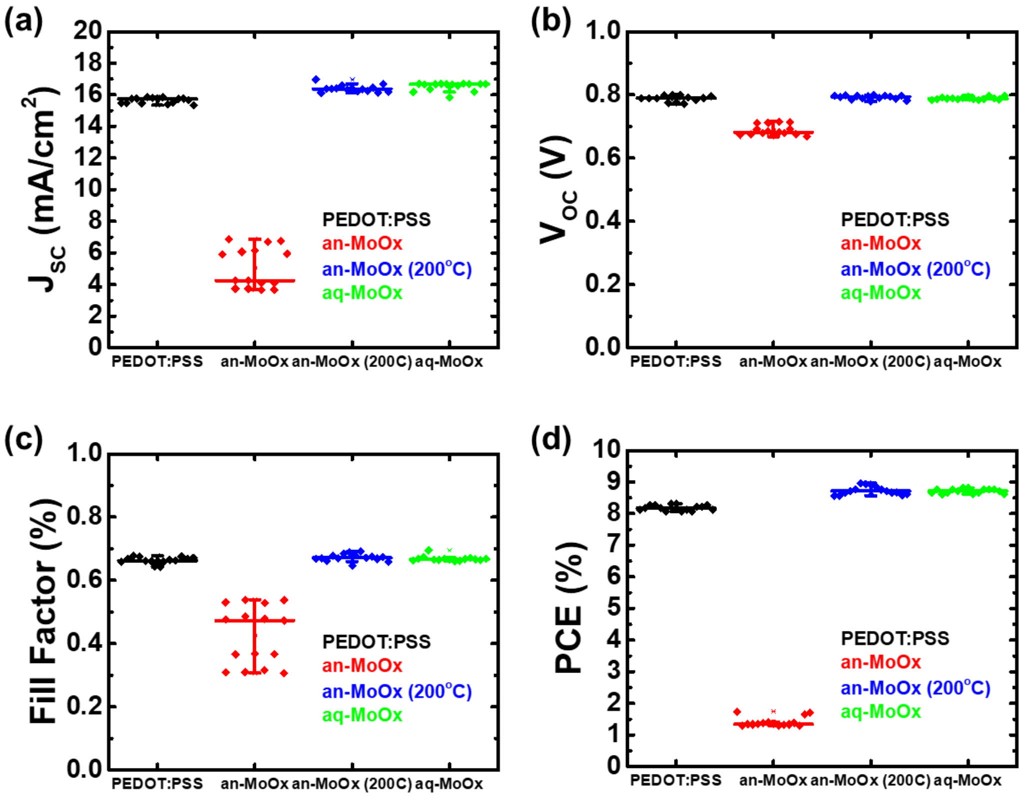

Figure S3. Statistics graph of a) J_{SC}, b) V_{OC}, c) FF, and d) PCE of the conventional polymer solar cell based on PTB7-Th:$PC_{71}BM$.

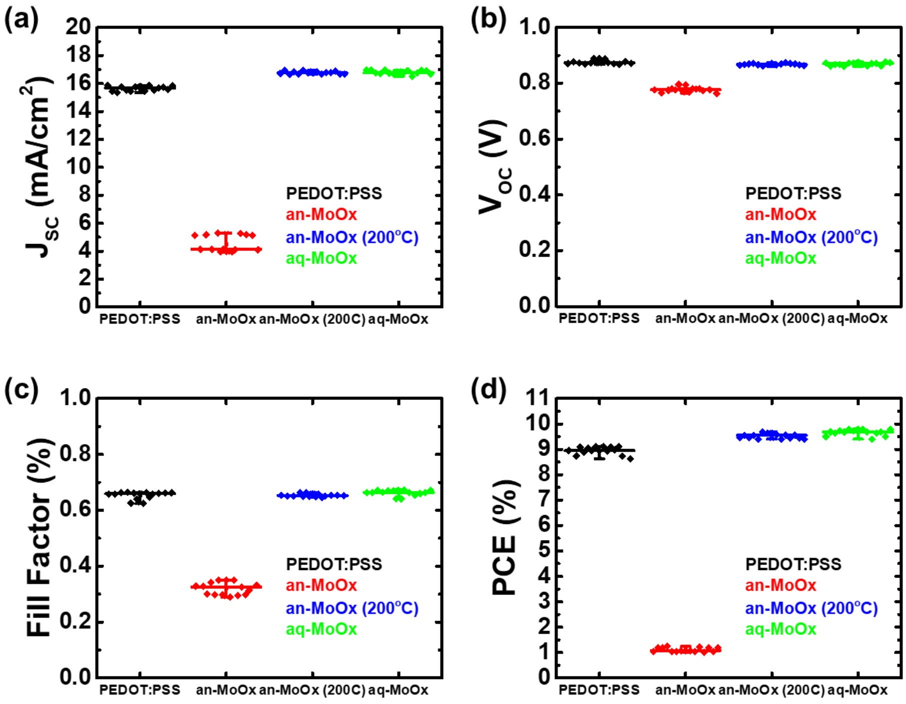

Figure S4. Statistics graph of a) J_{SC}, b) V_{OC}, c) FF, and d) PCE of the conventional polymer solar cell based on PBDTTPD-HT:IDIC.

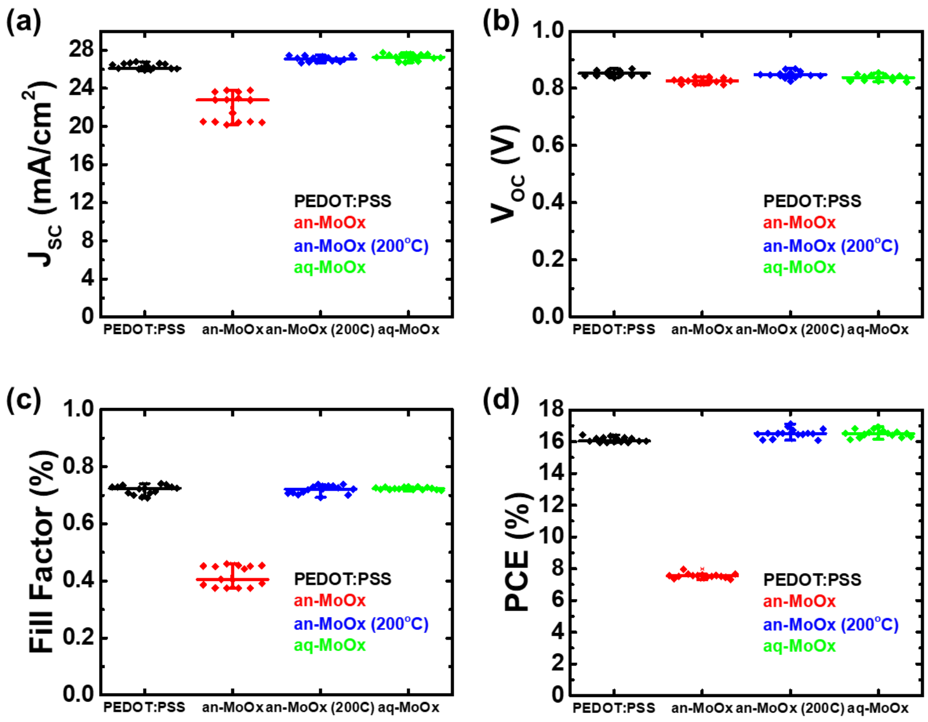

Figure S5. Statistics graph of a) J_{SC}, b) V_{OC}, c) FF, and d) PCE of the conventional polymer solar cell based on PBDB-T-2F:Y6.

Table S2. Photovoltaic performance of the conventional polymer solar cell based on the PBDB-T-2F:Y6 blend with an aqueous MoO_x film as a function of the annealing temperature.

Condition	J_{sc} [mA/cm^2]	V_{oc} [V]	FF [%]	Best PCE [%]
PEDOT:PSS	26.44	0.86	0.72	16.25
Aqueous MoO_x 50°C	27.40	0.84	0.71	16.27
Aqueous MoO_x 100°C	27.30	0.84	0.70	16.21
Aqueous MoO_x 150°C	27.28	0.85	0.70	16.19
Aqueous MoO_x 200°C	27.38	0.85	0.70	16.38

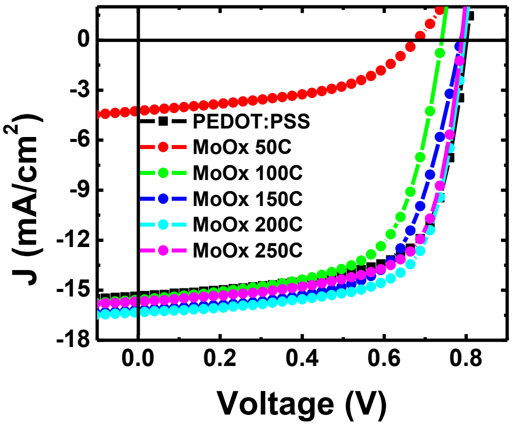

Figure S6. *J-V* characteristics of the conventional polymer solar cell based on PTB7-Th:$PC_{71}BM$ blend films with an anhydrous MoO_x film as a function of the annealing temperature.

Table S3. Photovoltaic parameters of the conventional polymer solar cell based on PTB7-Th:$PC_{71}BM$ with an anhydrous MoO_x film as a function of the annealing temperature.

Condition	J_{sc} [mA/cm^2]	V_{oc} [V]	FF [%]	Best PCE [%]
PEDOT:PSS	15.67	0.79	0.67	8.27
Anhydrous MoO_x 50℃	4.25	0.68	0.48	1.37
Anhydrous MoO_x 100℃	15.59	0.74	0.64	7.33
Anhydrous MoO_x 150℃	16.20	0.79	0.64	8.15
Anhydrous MoO_x 200℃	16.60	0.79	0.68	8.96
Anhydrous MoO_x 250℃	15.68	0.79	0.68	8.43

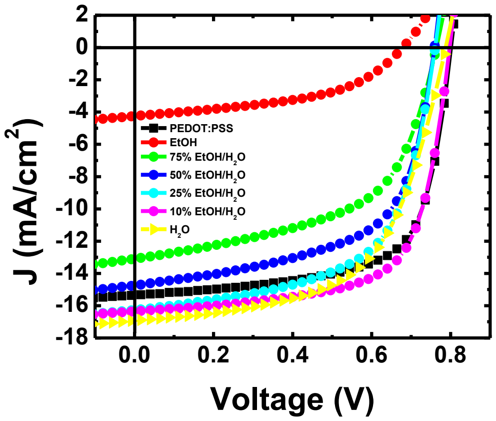

Figure S7. *J-V* characteristics of the conventional polymer solar cells based on PTB7-Th:$PC_{71}BM$ blend films with aqueous MoO_x with various H_2O concentrations.

Table S4. Photovoltaic performance of the conventional polymer solar cell based on PTB7-Th:$PC_{71}BM$ blend films with aqueous MoO_x with various H_2O concentrations.

Condition	J_{sc} [mA/cm^2]	V_{oc} [V]	FF [%]	PCE [%]
PEDOT:PSS	15.35	0.80	0.67	8.29
EtOH 100%	4.25	0.68	0.48	1.37
EtOH 75% / H_2O 25%	13.10	0.76	0.55	5.51
EtOH 50% / H_2O 50%	14.75	0.76	0.60	6.73
EtOH 25% / H_2O 75%	16.28	0.76	0.61	7.51
EtOH 10% / H_2O 90%	16.36	0.80	0.67	8.76
H_2O 100%	16.92	0.79	0.58	7.74

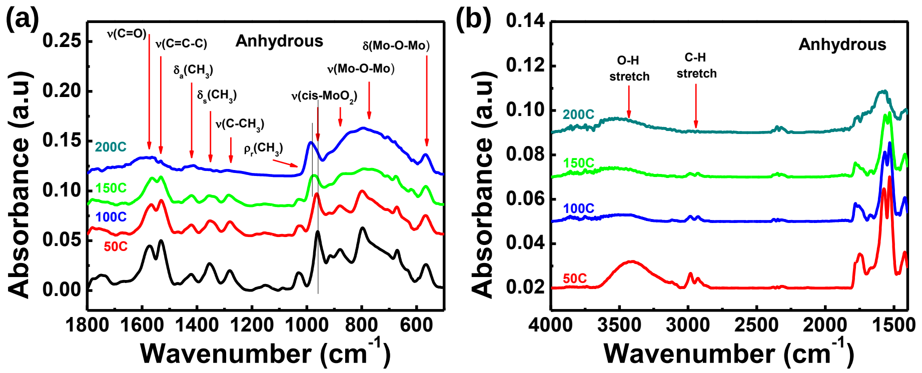

Figure S8. FTIR spectra obtained from anhydrous MoO_x layers annealed at various temperatures.

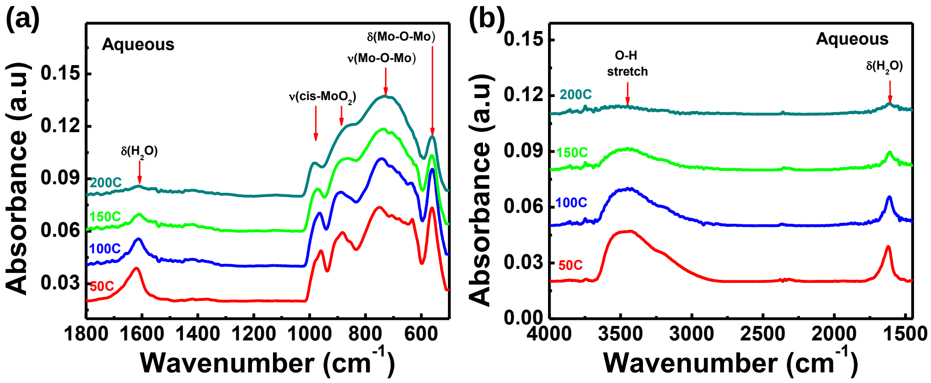

Figure S9. FTIR spectra obtained from aqueous MoOx layers annealed at various temperatures.

Table S5. The Mo^{6+}/Mo^{5+}, Oxygen-HBE/Oxygen-LBE, and O atom/Mo atom ratios calculated from the XPS data.

	Mo^{6+} / Mo^{5+}	Oxygen-HBE/Oxygen-LBE	Mo:O
Anhydrous MoO_x	3.86	1.28	1:2.80
Anhydrous MoO_x 200℃	5.13	0.41	1:2.99
Aqueous MoO_x	4.59	0.94	1:2.93

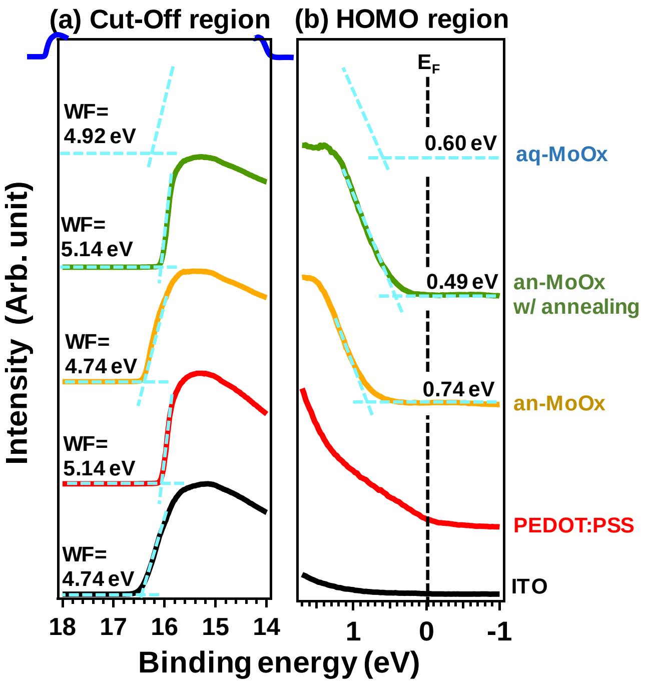

Figure S10. UPS spectra of MoO_x layers.

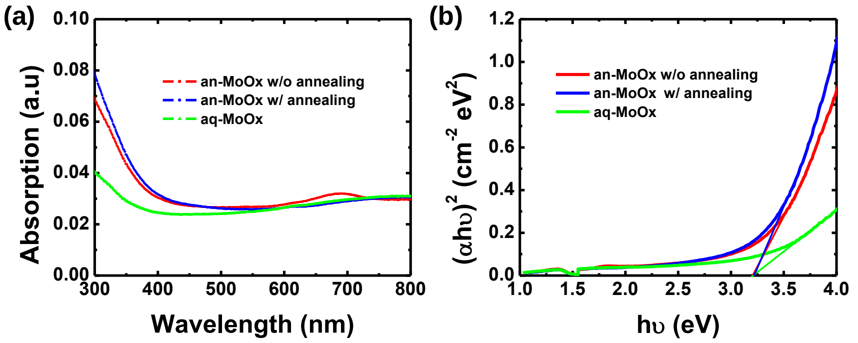

Figure S11. (a) Absorption spectra and (b) T_{auc} plot of MoO_x layers.

Table S6. Summary of the parameters obtained from UPS data for the MoO_x layers. The notations WF, E_g, IP, Φ_e, and Φ_h denote the work function, optical band gap, ion potential, electron injection barrier, and hole injection barrier, respectively.

Condition	WF (eV)	E_g (eV)	IP (eV)	ϕ_e (eV)	ϕ_h (eV)
ITO	4.74	N/A	N/A	N/A	N/A
ITO/PEDOT:PSS	5.14	N/A	N/A	N/A	N/A
ITO/an-MoO_x w/o annealing	4.74	3.22	5.48	2.48	0.74
ITO/an-MoO_x w/ annealing	5.14	3.22	5.63	2.73	0.49
ITO/aq-MoO_x	4.92	3.22	5.52	2.62	0.60

Table S7. The thickness of various hole transport layers as PEDOT:PSS, anhydrous MoO_x, and aqueous MoO_x.

Condition	Thickness (nm) [Average for 3 devices]
PEDOT:PSS	51.0 (±0.6)
Anhydrous MoO_x	14.9 (±0.2)
Anhydrous MoO_x (200℃)	16.4 (±0.3)
Aqueous MoO_x	13.7 (±0.4)

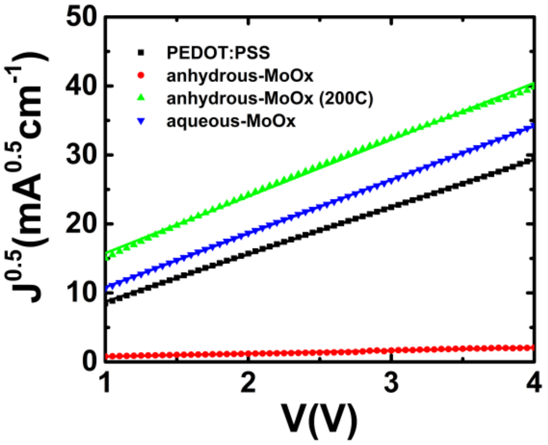

Figure S12. Hole mobilities of OPVs based on PBDB-T-2F:Y6 blend films with various hole transport layers as PEDOT:PSS, anhydrous MoOx and aqueous MoOx. The hole-only devices configuration is ITO/HTL/PBDB-T-2F:Y6/evaporated MoOx/Ag.

Table S8. Hole mobilities of OPVs based on PBDB-T-2F:Y6 blend films with various hole transport layers as PEDOT:PSS, anhydrous MoOx and aqueous MoOx.

Condition	Slope	μ_h ($cm^2.V^{-1}s^{-1}$)
PEDOT:PSS/PBDB-T-2F:Y6	6.87	$4.57x10^{-4}$
Anhydrous MoOx/PBDB-T-2F:Y6	0.44	$1.87x10^{-6}$
Anhydrous MoOx (200C)/PBDB-T-2F:Y6	8.22	$6.54x10^{-4}$
Aqueous MoOx/PBDB-T-2F:Y6	7.79	$5.87\ x10^{-4}$

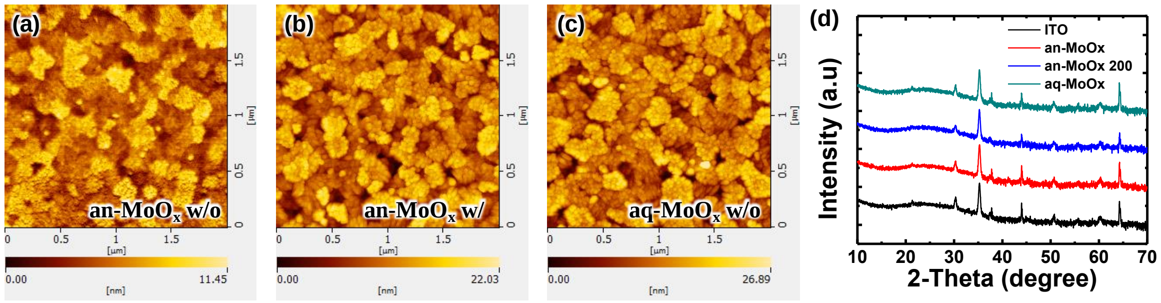

Figure S13. Atomic Force Microscope (AFM) images of (a) an-MoO_x without annealing, (b) an-MoO_x with annealing at 200 ^oC, and (c) aq-MoO_x without annealing, and (d) XRD results.

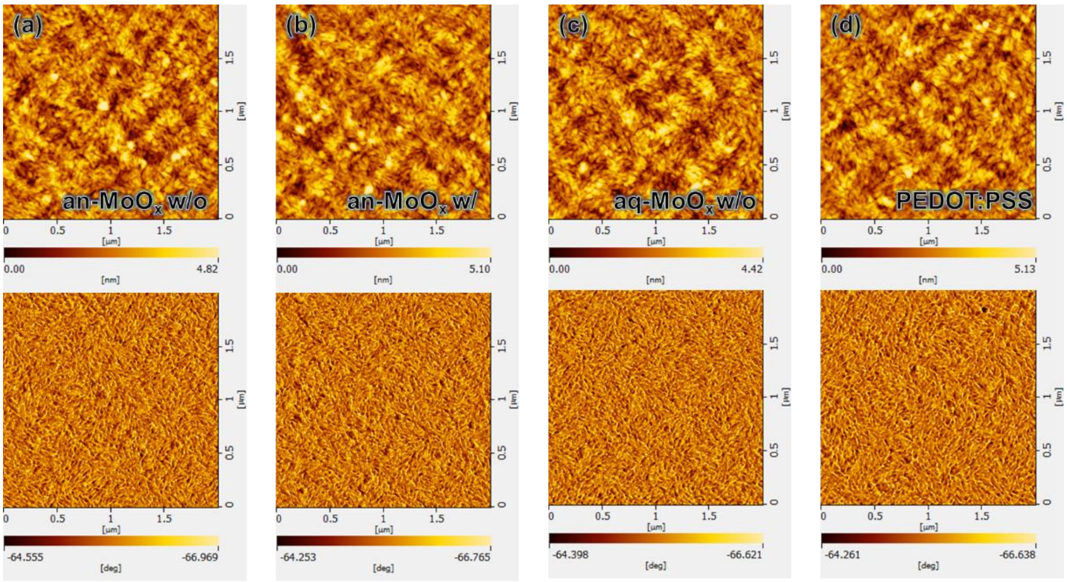

Figure S14. Atomic Force Microscope (AFM) topography images (top) and phase image (bottom) of PBDB-T-2F:Y6 active layer casted on (a) an-MoOx without annealing, (b) an-MoOx with annealing at 200 °C, (c) aq-MoOx without annealing, and (d) PEDOT:PSS.

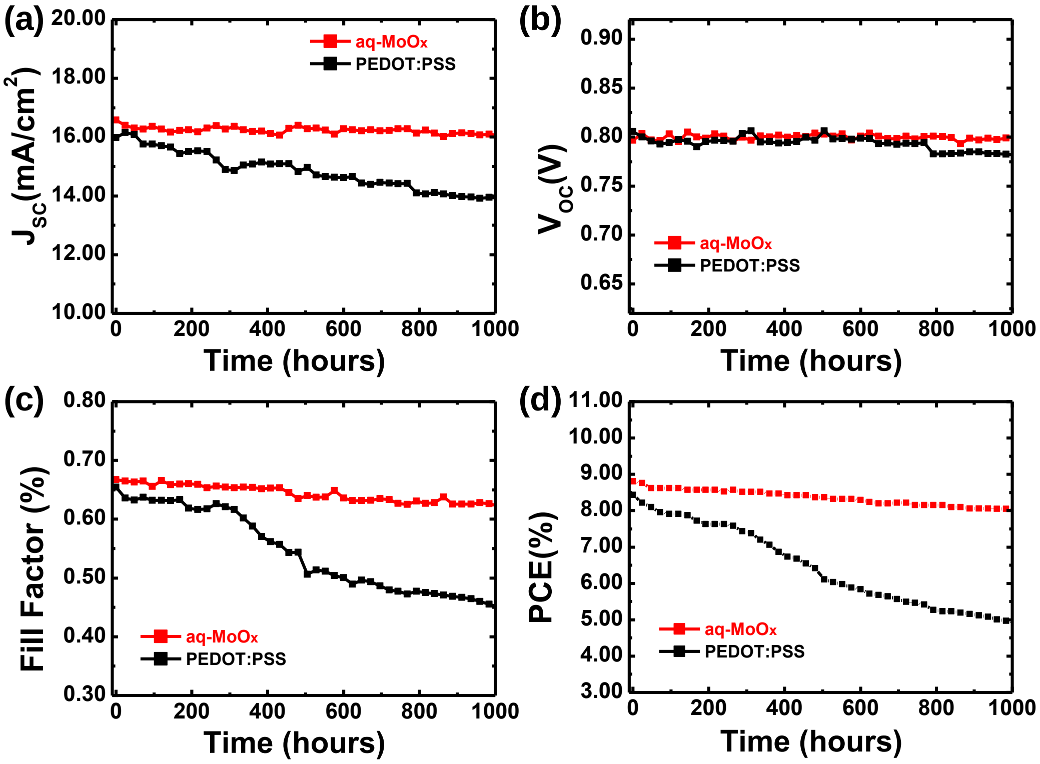

Figure S15. Storage stability performed for PTB7-Th:PC$_{71}$BM solar cells
